# Protein kinase C isoforms α, δ and ε are differentially expressed in mouse ovaries at different stages of postnatal development

**DOI:** 10.1186/s13048-014-0117-z

**Published:** 2014-12-10

**Authors:** Filiz Tepekoy, Ismail Ustunel, Gokhan Akkoyunlu

**Affiliations:** Department of Histology and Embryology, Faculty of Medicine, Akdeniz University, 07070 Campus, Antalya, Turkey

**Keywords:** Protein kinase C, Ovary, Postnatal development

## Abstract

**Background:**

The protein kinase C (PKC) is a family of serine/threonine kinases that consists of 12 different isoforms. Since PKC isoform expressions are known to be specific for different cell types and postnatal developmental stages, we aimed to determine immunolocalizations and protein expression levels of different PKC isoforms in pre-pubertal, pubertal and adult mouse ovaries.

**Methods:**

Ovaries were obtained from postnatal day 1 (PND1) and PND7 of pre-pubertal, PND21 of pubertal and PND60 of adult mice. Immunolocalizations of PKCα, PKCδ and PKCε isoforms were determined and immunostainings in different cellular components of all follicular stages were evaluated by H-Score. PKCα, PKCδ and PKCε protein expression levels were determined by Western blot. The bands were quantified via ImageJ software. The data obtained from H-Score and ImageJ evaluations were analyzed by ANOVA statistical test.

**Results:**

PKCα immunostainings were more intense in oocytes when compared to granulosa and theca cells at different follicular stages of all groups. The Western blot analysis revealed that PKCα expression was significantly higher in PND60 adult ovaries. Conversely, PKCδ immunostainings were more intense in granulosa cells. According to the Western blot analysis, PKCδ protein expression was also higher in PND60 and significantly lower in PND1 ovaries. PKCε immunostaining was more apparent in oocytes. PKCε protein expression was significantly higher in adult PND60 and pubertal PND21 ovaries when compared to pre-pubertal PND7 and PND1 ovaries. Interestingly, PKCε immunostaining was significantly higher in primordial follicles, though PKCα and PKCδ immunostainings were more apparent in larger follicles. PKCα immunostainings of corpora lutea (CL) were significantly higher when compared to follicles in PND60 ovaries.

**Conclusions:**

This study demonstrates that PKCα, PKCδ and PKCε isoforms are differentially expressed in particular cellular components of pre-pubertal, pubertal and adult mouse ovarian follicles. Therefore, we suggest that each PKC isoform has unique functions that are controlled by gonadotropin dependent mechanisms during follicular growth, oocyte maturation, ovulation and luteinization.

## Background

Main ovarian functions related to female fertility include folliculogenesis, oocyte maturation, ovulation and luteinization processes consecutively controlled by gonadotropin induced signal transduction pathways. The activation of these pathways shows difference between pre-pubertal, pubertal and adult ovaries due to variation of gonadotropin levels.

As reviewed by Richards J. et al. [1], particular signaling components show variable expression levels in ovarian follicles at different stages and corpus luteum (CL). Though, a number of signaling pathways are known to be specific for different stages of follicular development, less is known about protein kinase C (PKC) expression levels at particular follicular stages in pre-pubertal, pubertal or adult ovaries.

PKC is a family of serine/threonine kinases that play essential roles in many signal transduction pathways [2,3]. PKC family consists of 12 different isoforms that show difference in terms of amino acid sequences of specific domains [4]. These isoforms are classified into 3 subtypes based on allosteric activators [5]: (a) conventional isoforms that are activated by Ca^2+^ and diacylglycerol (DAG) (PKC α, β1, β2, and γ), (b) novel isoforms that are activated by DAG for activation (PKC μ, η, θ, ε and δ), and (c) atypical isoforms that are activated independently of Ca^2+^ or DAG (PKC ι, λ and ζ) [6]. Ca^2+^ and DAG serve as allosteric activators of PKC as they bind to regulator domains of PKC for activation [7]. PKC isoform expressions show specifity for the cell types and developmental stages [8]. PKC activation via specific hormones or phorbol ester leads to translocation of some of the PKCs to new subcellular sites where they phosphorylate their specific target proteins [9]. Earlier studies documented the importance of PKC in several physiological processes in ovary such as granulosa cell proliferation for follicular growth [10], oocyte maturation [11], ovulation [6] and luteinization [12].

During follicular growth, controlled division of hamster granulosa cells is activated by a self-sustaining loop of PKC-MAPK-PLA2G4 (protein kinase C-mitogen activated protein kinases-phospholipase A2 group IV family) triggered by FSH-EGF-EGFR kinase (follicle stimulating hormone- epidermal growth factor-epidermal growth factor receptor kinase) pathway [10]. Self-sustaining loop including PKC is activated by FSH via triggering EGF and EGFR kinase respectively. EGFR kinase phosphorylates MAPK3/1 by sequential activation of RAF1 and MAP2K1. PKC is stimulated by PLA2G4 after MAPK3/1 activation and self-sustaining loop also becomes activated. After 2 h of exposure to FSH or EGF, self-sustaining loop becomes independent of the receptor kinase and sustains MAPK3/1 activity leading to cyclin dependent kinase 4 (CDK4) activation and DNA synthesis [10].

Isoform specifity of PKC family during follicular development is also underlined in earlier studies. PKCδ is found to be associated with anti-apoptotic actions of basic fibroblast growth factor (bFGF) sustaining rat granulosa cell viability [13]. bFGF’s anti-apoptotic activity is suggested to be controlled by maintaining [Ca^2+^]_i_ within a physiological range [13]. PKC inhibitor chelerythrine chloride is shown to attenuate bFGF’s ability to regulate [Ca^2+^]_i_ in granulosa cells [13]. Besides, PKCδ-specific inhibitor, rottlerin, abrogates bFGF’s anti-apoptotic action whereas an activator of PKC, 12-O-Tetradecanoylphorbol-13-acetate (TPA-also known as phorbol 12-myristate 13-acetate [PMA]), prevents granulosa cell apoptosis [13]. Additionally, various PKC isoforms are expressed specifically at different stages of mouse oocyte maturation process. The conventional PKC (cPKC) isoforms, PKCα, PKC-βI, and PKC-βII are expressed in mouse oocytes at germinal vesicle (GV) and metaphase II (MII) stages. Treatment of GV oocytes with PKC activator TPA, resulted in PKCα accumulation at the plasma membrane, whereas treatment of MII oocytes with TPA leads to PKCβ accumulation in addition to PKCα accumulation at the plasma membrane [14]. In addition to conventional isoforms, novel (δ) and atypical (λ and ζ) isoforms are shown to be expressed in prophase I and MII stage mouse oocytes [15,16]. PKCδ is located in the cytoplasm, associated with the spindle apparatus during the first meiotic division, whereas in MII stage oocytes PKCδ is found to be in a speckled pattern, associated with the chromosomes and upon oocyte activation, the protein is dephosphorylated and accumulates in the nuclei of early mouse embryos [11]. Global activation of PKC via PMA during bovine oocyte maturation is related to an acceleration of nuclear maturation [17]. In a follicle culture model, it is suggested that cPKC isoforms PKCα and PKCβI participate in FSH-induced meiotic resumption of mouse follicle enclosed oocytes, possibly by the activation of EGFR [18]. EGF and Amphiregulin are reported to reverse the inhibitory effects of PKCα and βI inhibitor Gö6976 on FSH-induced meiosis resumption indicating that PKCα and βI participate in FSH-induced oocyte meiotic resumption possibly by EGF and/or EGF-like factors [19]. EGF-like factors released from granulosa cells affect stimulation of ERK1/2MAPK dependent gene transcription related to ovulation in cumulus cells. The downstream positive signal that triggers germinal vesicle break-down (GVBD) is suggested to be related to reduced levels of cGMP transmission to the oocyte from cumulus cells. The reduction of cGMP transmission causes phosphodiesterase 3A (PDE3A) activation and thus cAMP is hydrolyzed by PDE3A which stops meiotic arrest and results in resumption of meiosis. Though it is suggested that, the effects on cGMP may be dependent on activation of the EGF network [20,21], direct relation of this pathway with PKC remains unknown.

PKC is also related with control of ovulation. PKCζ, activation by luteinizing hormone (LH) or forskolin, results in the induction of transcription factor nerve growth factor-induced protein B in cultured granulosa cells of pre-ovulatory follicles [12]. In primary mouse granulosa cells, PKC activation via PMA treatment leads to the induction of ovulatory *Prkg2* gene through stimulation of extracellular signal-regulated kinase (ERK) [6].

The effectiveness of PKC in luteal cells of CL is also documented. In rat luteal cells, activation of the MAPK signaling cascade is mimicked by PKC activator PMA suggesting that PKC activates MAPK signaling in CL [22]. When PKCε is inhibited with PKCε specific inhibitors, prostaglandin F2 alpha activity in reduction of progesterone synthesis/ secretion is also inhibited in bovine luteal cells [23]. PKC activator PMA is shown to mimic the effect of adenosine triphosphate (ATP) by reducing the human chorionic gonadotropin (hCG)-induced cyclic adenosine monophosphate (cAMP) production in human granulosa-luteal cells. The inhibitory action of ATP in hCG evoked cAMP production is reversed by global (Staurosporin), and selective (Bisindolylmaleimide I for PKCα, -β, and -γ), inhibitors of PKC [24].

PKC is included in the signal transduction pathways that control folliculogenesis, ovulation and luteinization under the control of specific hormones and growth factors. Considering the fact that, the effectiveness of these hormones and growth factors at pre-pubertal, pubertal and adult ovaries shows variation, we hypothesize that, PKC is differentially expressed in mouse ovaries at different stages of postnatal life and the expression levels of PKC isoforms in particular cell types of ovarian follicles at different stages and luteal cells of CL show variation which may be due to isoform specific functions of PKC in the ovary.

## Methods

### Animals and tissue processing

Female (n = 12, 3 for each group) Balb/C intact mice supplied by Animal Care and Usage Comittee of Akdeniz University were maintained under standard laboratory conditions (21 ± 1°C; ambient temperature; controlled light/dark conditions, 14 L: 10D) and were given food and water ad libitum. The day of birth was designated as PND0 and the experimental protocol was approved by the Animal Ethics Committee of Akdeniz University, Turkey (2009.08.36).

Ovaries were harvested from postnatal days (PND) 1, 7, 21 and 60 female mice and left ovary samples were fixed by immersion in Bouin’s fixative (75 mL of saturated aqueous solution of picric acid [Sigma-Aldrich Co. LLC, Steinheim, Germany], 25 mL of formalin [Merck, NJ, USA] and 5 mL of glacial acetic acid [Sigma-Aldrich Co. LLC, Steinheim, Germany]) at room temperature for 4 hours. Then tissues were dehydrated through a graded series of ethanol, cleared with xylene and finally embedded in paraffin wax for immunohistochemical investigations. Right ovary samples were preserved in -80°C freezer until they were processed for protein extraction for Western blotting.

### Immunohistochemistry

Paraffin-embedded samples were cut into 5 μm sections and placed on superfrost ultra plus adhesion slides, (Thermo Sci., Rockford, IL, USA). After deparaffinization, the slides were boiled in citrate buffer (pH: 6.0) for 10 minutes for antigen retrieval and cooled for 20 minutes at room temperature. Then, the slides were immersed in 3% hydrogen peroxide for 20 minutes to block endogenous peroxidase. They were then incubated in a humidified chamber with UltraV block (Lab-vision, Fremont, CA, USA) for 7 minutes at room temperature. Excess serum was drained and the slides were incubated with rabbit polyclonal primary antibodies PKCα (sc-208) PKCδ (sc-937) and PKCε (sc-214) antibodies (Santa Cruz, CA, USA) at 1:250 dilution overnight in a humidified chamber at 4°C. Negative controls were performed by replacing the primary antibody with phosphate buffered saline (PBS). The slides were washed three times for 5 minutes with PBS and then incubated with peroxidase-conjugated anti-rabbit secondary antibody (Vector Lab. Inc., Burlingame, CA, USA) at 1:500 dilution for 30 minutes at room temperature. Then the slides were washed with PBS and peroxidase activity was visualized with 3,3’-Diaminobenzidine (DAB) (Sigma-Aldrich Co. LLC, Steinheim, Germany) for 3–5 minutes. The slides were counterstained with hematoxylin, dehydrated, mounted in entellan (Merck, NJ, USA) and examined by light microscopy. Since, immunohistochemical observations revealed only the localization of the proteins, semi-quantitative analysis of the observations performed in order to present differential amount of the proteins in specific cell types.

### SDS polyacrylamide gel electrophoresis and Western blotting

In order to perform protein extraction and immunoblot analysis of PKCα, PKCδ and PKCε; tissues were weighed and put into homogenization buffer (10 mM Tris–HCL, 1 mMEDTA, 2.5% SDS, 1 mM phenylmethylsulfonylfluoride, 1 μg/mL leu-peptin) supplemented with CompleteR protease inhibitor cocktail (Boehringer, Mannheim, Germany). After homogenization, samples were centrifuged at 10,000 × g for 10 min. Supernatants were collected and stored at −80°C. The protein concentration was determined by Lowry assay [25] and 50 μg protein was applied per lane. Prior to electrophoresis, samples were boiled for 5 min at 95°C. Samples were subjected to SDS polyacrylamide gel electrophoresis and then were transferred onto nitrocellulose membranes (Amersham Pharmacia, Piscat-away, NJ, USA) in a buffer containing 0.2 mol/l glycine, 25 mMTris and 20% methanol overnight. Successful transfer was confirmed by Ponceau S (Sigma-Aldrich Co. LLC, Steinheim, Germany) staining of the blots. The membranes were blocked for 1 h with 5% non-fat dry milk (BioRad, Hercules, CA, USA) and 0.1% Tween 20 (Sigma-Aldrich Co. LLC, Steinheim, Germany) in 0.14 mol/l Trisbuffered saline (TBS) pH:7.2–7.4 at 4°C. Blotting membranes were incubated overnight at 4°C with PKCα (sc-208) PKCδ (sc-937) and PKCε (sc-214) antibodies (Santa Cruz, CA, USA) at 1:1000 dilution. After washing steps, the membranes were further incubated with goat anti rabbit IgG horseradish peroxidase conjugate (BioRad, Her-cules, CA, USA) diluted 1:5000 for 1 h at room temperature. Immunolabeling was visualized using the chemiluminescence based SuperSignal CL HRP Substrate System (Pierce, Rockford, IL, USA) and the membranes were exposed to Hyperfilm (AmershamPharmacia). β-Actin antibody (1:5000 dilution) (Abcam, Cambridge, UK) was used as an internal control for each blotting in order to confirm the equal loading of the samples. The bands were quantified using NIH image analysis software (ImageJ Version 1.36b, National Institutes of Health, Bethesda, MD, USA). During Western blot experiments, for all isoforms, multiple bands were detected within the predicted molecular weight range, however there was only one distinct band for all isoforms. When analyzing Western blot bands, distinct bands were evaluated and the other insignificant bands were considered to be specific for different PKC isoforms. Therefore, those insignificant bands were excluded from the Western blot analysis.

Since this technique revealed the total amount of PKC isoforms in whole ovaries, differential expressions of these proteins in different cell types of the ovaries were evaluated by semi-quantitative analysis of immunohistochemical observations.

### Histologic score (H-SCORE) and statistical analysis

The evaluations of the immunohistochemical labeling for PKCα, PKCδ and PKCε in postnatal ovaries were accomplished by utilizing H-SCORE as it has been used in previous studies [26]. Briefly, immunohistochemical labelling for PKCα, PKCδ and PKCε was evaluated in a semiquantitative fashion by eye on a scale of 0 [absent] to 3 [most intense]. For each slide, an H-SCORE value was derived by summing the percentages of labelled cells at each labeling intensity, multiplied by the weighted intensity of the labeling, that is HSCORE = ∑Pi(i + 1), where i defines the intensity score and Pi is the corresponding percentages of the cells labeled. For each slide, 10 different areas were evaluated using the light microscope at 400× magnification, and the percentage of the cells for each labeling intensity within these areas was determined at different times by two investigators (FT and GA) blinded to the source and type of the tissues. The average score of the two was used.

H-SCORE value for each follicle is obtained from the averages of H-SCORE values of oocytes and granulosa cells. The immunohistochemistry data from the H-SCORE and Western blotting data from ImageJ were analyzed with non-parametric ANOVA on ranks (Kruskal–Wallis test) and parametric One-way ANOVA, Holm Sidak method. The values were presented as mean ± SEM. Statistical calculations were performed using Sigma Stat for Windows, version 3.0 (Jandel Scientific Corp. San Rafael, CA, USA). Statistical significance was defined as *P* <0.05.

## Results

### Immunolocalization of PKCα in mouse ovaries

**Figure 1:**
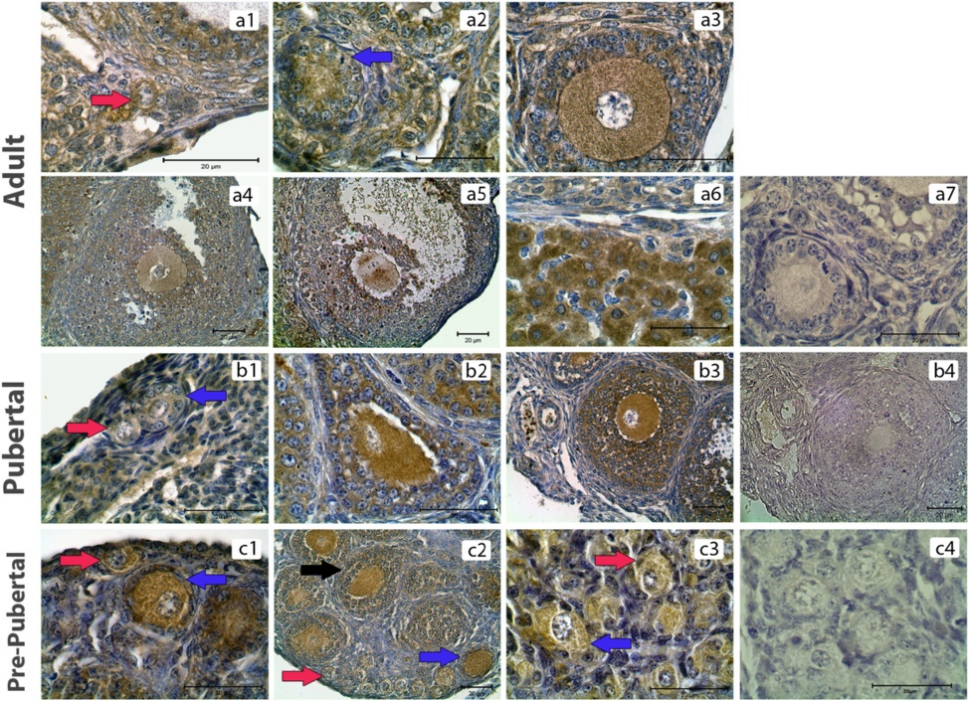
**PKCα immunostainings in postnatal mouse ovaries. (a1-a6)** PND60 ovary primordial (a1), primary (a2), secondary (a3), pre-antral (a4), antral (a5) follicles and CL (a6). **(b1-b3)** PND21 ovary primordial and primary (b1), secondary (b2), pre-antral (b3) follicles. **(c1-c3)** PND7 ovary primordial and primary (c1), secondary (c2) follicles. PND1 ovary (c3). a7, b4 and c4 are representative negative control sections. Counterstain hematoxylin. Red arrows: Primordial follicles, Blue arrows: Primary follicles. Black arrow: Secondary follicle.

**Figure 2:**
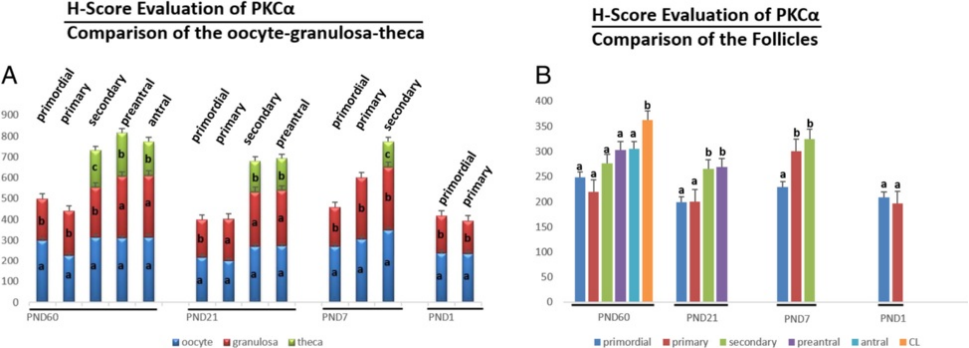
**H-Score evaluations of PKCα stainings in postnatal mouse ovaries in terms of follicular cells (A) and follicular stages (B).** Different letters mark statistical significance (p < 0.05). (One way anova, Holm Sidak method).

PKCα was detected in the cytoplasm of granulosa cells, theca cells and oocytes of all groups and luteal cells of PND60 ovaries (Figure 1). H-Score evaluation revealed that PKCα immunostaining was intense in oocytes of all follicles (Figure 2A). Higher PKCα immunostainings in oocytes of primordial (PND60, PND7 and PND1), primary (PND60, PND7 and PND1) and secondary follicles (PND60 and PND7) in adult and pre-pubertal ovaries were statistically significant. However, in PND21 ovaries, only oocytes of primordial follicles had significantly higher immunostaining of PKCα. Significantly higher immunostaining of PKCα was detected in CL when compared to follicles at different stages of development in PND60 ovaries. Also, PKCα immunostaining was decreased in primary and primordial follicles when compared to antral, preantral and secondary follicles in PND60 ovaries (Figures 1.a1-a7, 2B). In PND21 ovary, primordial and primary follicle PKCα immunostaining levels were significantly lower than the immunostaining levels in secondary and preantral follicles (Figures 1.b1-b4, 2B). In PND7 ovary, PKCα immunostaining level in primordial follicles was significantly lower than primary and secondary follicles (Figures 1.c1-c2, 2B). In PND1 ovary, though primordial follicle PKCα immunostaining level was higher than primary follicle (Figures 1.c3, 2B), this difference was not significant.

### Immunolocalization of PKCδ in mouse ovaries

**Figure 3:**
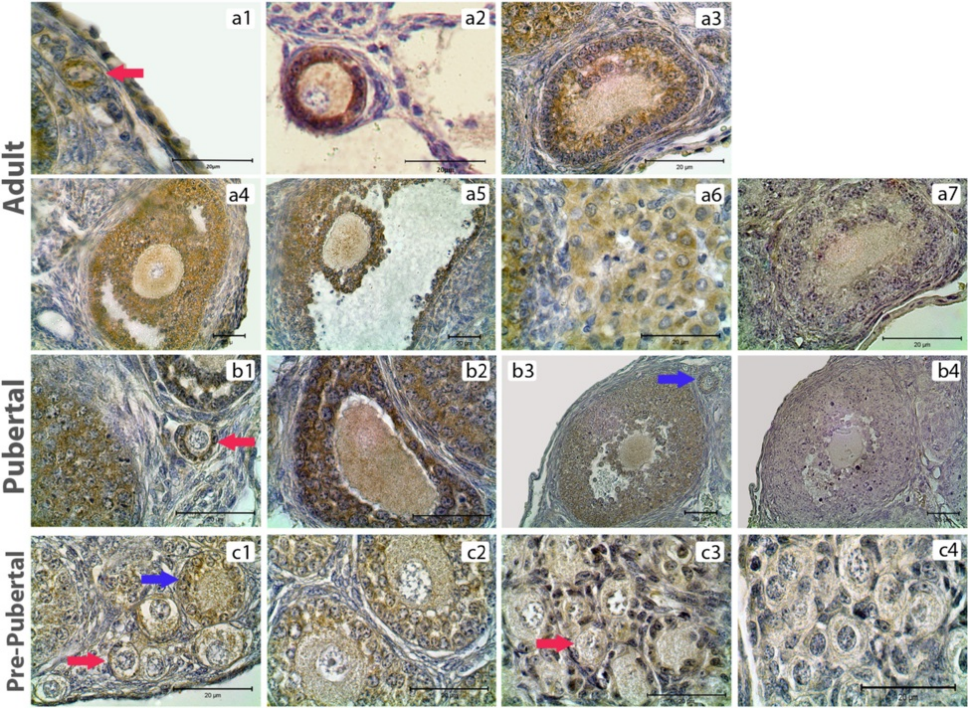
**PKCδ immunostainings in postnatal mouse ovaries. (a1-a6)** PND60 ovary primordial (a1), primary (a2), secondary (a3), pre-antral (a4), antral (a5) follicles and CL (a6). **(b1-b3)** PND21 ovary primordial and primary (b1), secondary (b2), pre-antral (b3) follicles. **(c1-c3)** PND7 ovary; primordial and primary (c1), secondary (c2) follicles, PND1 ovary (c3). a7, b4 and c4 are representative negative control sections. Counterstain hematoxylin. Red arrows: Primordial follicles, Blue arrows: Primary follicles.

**Figure 4:**
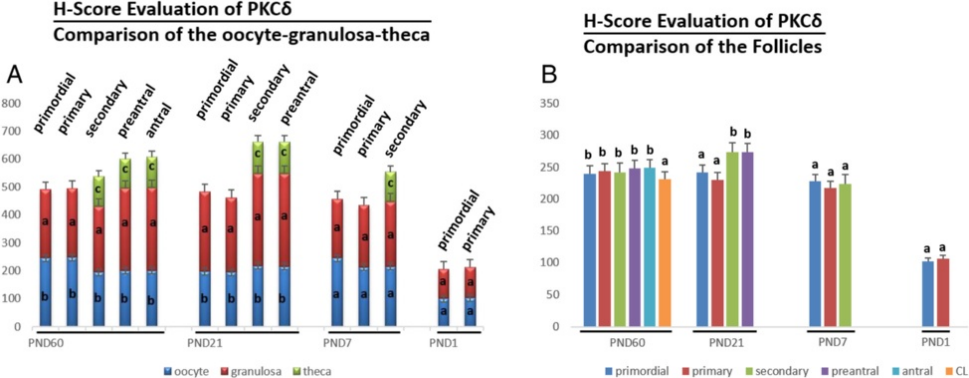
**H-score evaluations of PKCδ stainings in postnatal mouse ovaries in terms of follicular cells (A) and follicular stages (B).** Different letters mark statistical significance (p < 0.05). (One way anova, Holm Sidak method).

PKCδ was detected in the cytoplasm of granulosa cells, theca cells and oocytes of all groups and luteal cells of PND60 ovaries (Figure 3). H-Score evaluation revealed that PKCδ immunostaining was significantly enriched in granulosa cells of all follicles when compared to oocytes, theca cells and luteal cells (Figure 4A). Though higher PKCδ immunostaining levels were detected in preantral and antral follicles of PND60 ovaries, this difference was not statistically significant. However, PKCδ immunostaining was significantly lower in CL when compared to the follicles at different stages of development in PND60 ovaries (Figures 3.a1-a7, 4B). In PND21 ovary, primordial and primary follicle PKCδ immunostaining levels were significantly lower than the immunostaining levels in secondary and preantral follicles (Figures 3.b1-b4, 4B). In PND7 (Figures 3.c1-c2, 4B) and PND1 (Figures 3.c3, 4B) ovaries, there wasn’t any statistical difference in terms of PKCδ immunostaining levels between follicles. Interestingly, PKCδ immunostainings of granulosa cells were not significantly higher than oocytes of follicles of pre-pubertal ovaries, though PKCδ immunostaining in granulosa cells was significantly higher in PND60 and PND21 ovaries.

### Immunolocalization of PKCε in mouse ovaries

**Figure 5:**
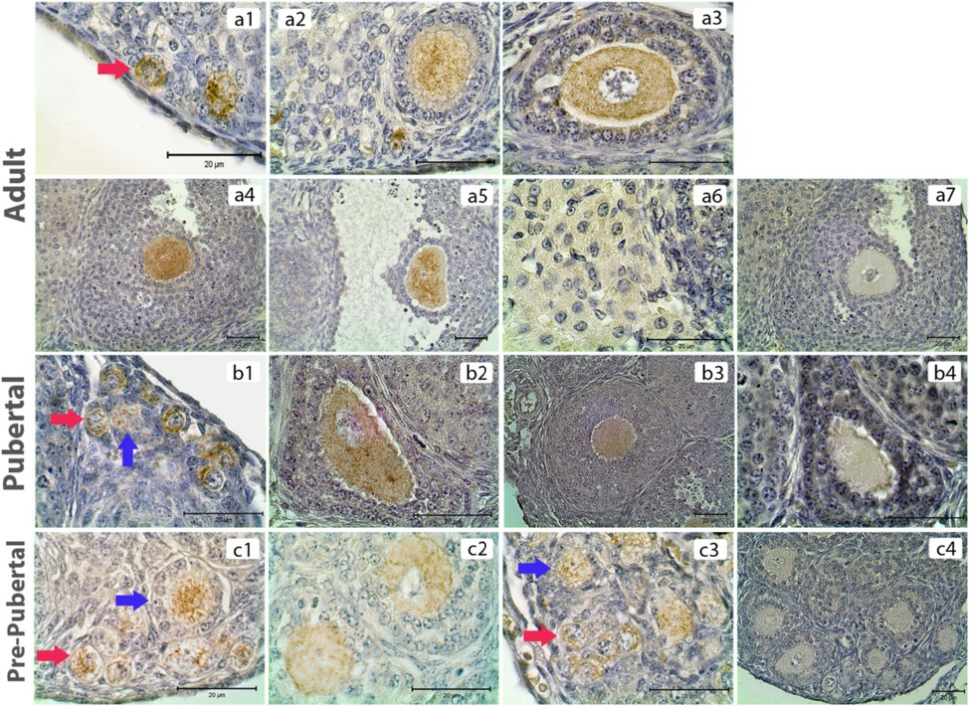
**PKCε immunostainings in postnatal mouse ovary. (a1-a6)** PND60 ovary primordial (a1), primary (a2), secondary (a3), pre-antral (a4), antral (a5) follicles and CL (a6). **(b1-b3)** PND21 ovary primordial and primary (b1), secondary (b2), pre-antral (b3). **(c1-c3)** PND7 ovary primordial and primary (c1), secondary (c2) follicles, PND1 (c3). a7, b4 and c4 are representative negative control sections. Counterstain hematoxylin. Red arrows: Primordial follicles, Blue arrows: Primary follicles.

**Figure 6:**
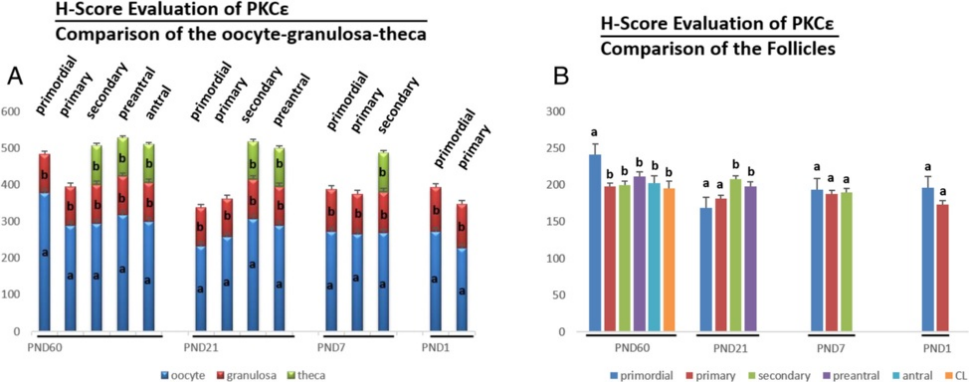
**H-Score evaluations of PKCε stainings in postnatal mouse ovaries in terms of follicular cells (A) and follicular stages (B).** Different letters mark statistical significance (p < 0.05). (One way anova, Holm Sidak method).

PKCε immunostaining was apparent only in the cytoplasm of oocytes of all groups (Figure 5). H-Score evaluation revealed that PKCε immunostaining was significantly lower in granulosa cells and theca cells of all follicles and also luteal cells when compared to oocytes (Figure 6A). Interestingly, significantly higher immunostaining of PKCε was detected in primordial follicles in PND60 ovaries (Figures 5.a1-a7, 6B). In PND21 ovary, primordial and primary follicle PKCε immunostaining levels were significantly lower than the immunostaining levels in secondary and preantral follicles (Figure 5.b1-b4). In PND7 (Figures 5.c1-c2, 6B) and PND1 (Figures 5.c3, 6B) ovaries, there wasn’t any statistical difference in terms of PKCε immunostaining levels between follicles though primordial follicles had the highest immunostaining level of PKCε in both groups.

### Western blot analysis of PKCα, PKCδ and PKCε in mouse ovaries

**Figure 7:**
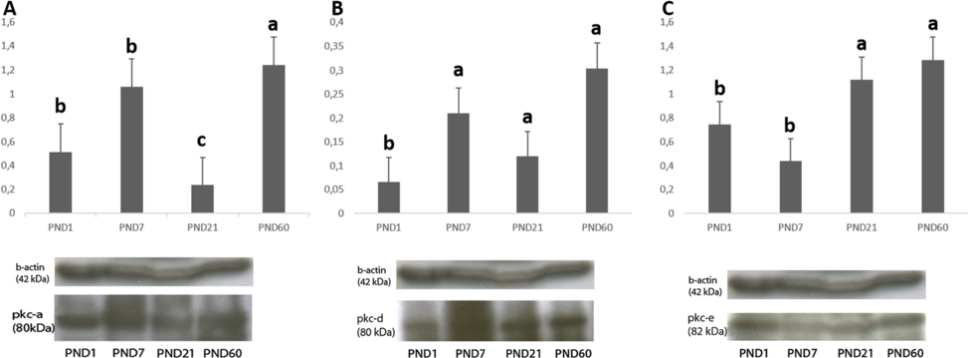
**Westen blot bands and graphics of mathematical values of ImageJ evaluations of PKCα (A), PKCδ (B) and PKCε (C) protein expressions in postnatal mouse ovaries.** Different letters mark statistical significance (p < 0.05). (One way anova, Holm Sidak method).

PKCα protein expression level was detected to be significantly higher in PND60 ovaries. PKCα expression in PND7 and PND1 was also significantly higher when compared to PND21 ovaries. The lowest PKCα protein expression was observed in PND21 ovaries (Figure 7A).

PKCδ expression was significantly lower in PND1 ovaries when compared to other groups. PND60 had the highest expression level of PKCδ (Figure 7B).

PKCε expression level was significantly higher in PND60 and PND21 ovaries when compared to pre-pubertal PND7 and PND1 ovaries (Figure 7C).

## Discussion

This study demonstrates that different PKC isoforms are differentially expressed in particular cellular components of ovarian follicles of pre-pubertal, pubertal and adult mouse ovaries.

Data obtained from H-Score evaluation of immunohistochemistry findings revealed that PKCα expression was more apparent in oocytes of all follicles in pre-pubertal, pubertal and adult ovaries, though it was also expressed in granulosa cells. Interestingly, in adult and pre-pubertal ovaries, intense immunostaining of PKCα in oocytes was statistically significant in primordial (PND60, PND7 and PND1), primary (PND60, PND7 and PND1) and secondary follicles (PND60 and PND7), whereas in PND21 ovaries only oocytes of primordial follicles had significantly higher immunostaining level of PKCα. These findings support the idea that PKCα expression in oocytes of larger follicles may have low significance due to granulosa-oocyte interactions during initiation of hormone dependent follicular growth [27,28]. PKCα immunostaining was shown to be higher in larger follicles in all groups which might be related to proliferation of granulosa cells and development of oocytes. Only in PND1 ovary, primordial follicles had higher immunostaining level of PKCα which was not statistically significant. PKCα protein expression showed the highest level in PND60 ovaries according to our Western blot experiments which might be due to its higher expression in larger follicles and CL.

Oocyte maturation of preovulatory follicles is induced by the signals from the extra-follicular environment during the LH surge and these signals are transmitted by cumulus cells to the oocyte through gap junctions as reviewed by Huang, Z. *et al*. [29]. Once the signal for cAMP reduction is received by cumulus cells, cAMP levels also decrease in oocytes through the action of PDE3A and results in protein kinase A (PKA) inactivation. Consequently, inactivating phosphorylation of CDC25b (cell division cycle 25b) by PKA is inhibited and active CDC25b removes inactivating phosphates from CDK1 of maturation promoting factor (MPF). Then MPF becomes active and maturation progresses [30]. While cumulus cells transmit signals to control oocyte maturation, oocyte secretes factors that promote follicle growth such as growth differentiation factor 9 (GDF9) and bone morphogenetic protein 15 (BMP15) [31].

The resumption of meiosis and entry into metaphase I (MI) is inhibited in oocytes arrested at prophase I upon PKC activation [16,32] whereas in *Xenopus*, mouse and rat MII oocytes, PKC activation promotes entry into interphase [33-35]. However, activation of PKC during bovine oocyte maturation is reported to induce GVBD [17]. In the present study, higher PKCα immunostaining level in oocytes of smaller follicles supports the idea that PKCα might be critical for meiotic arrest of oocytes rather than meiotic resumption.

According to the findings of the current study, another conventional PKC isoform, PKCδ was found to be expressed highly in granulosa cells of all follicles in pre-pubertal, pubertal and adult ovaries. Interestingly, intense immunostaining of PKCδ in granulosa cells compared to oocytes was significant only in adult and pubertal ovaries. This might be due to altered gonadotropin levels and related signal transduction pathways during and after puberty [28]. PKCδ immunostaining was also higher in larger follicles of all groups. Analysis of the Western blot bands showed that PKCδ had an increased expression in PND60 ovaries whereas PND1 ovaries had the lowest PKCδ expression level. These results might be related to higher expression of PKCδ in larger follicles.

The expression of PKCα and PKCδ in granulosa cells of all follicles might be related to their importance in granulosa cell proliferation and viability. The controlled division of hamster granulosa cells has already been found to be activated by a self-sustaining loop of PKC and MAPK pathway [10]. Though PKC is found to be included in activation of DNA synthesis through induction of MAPK pathway, it remains unknown which PKC isoform specifically participates in this process. PLA2G4 which takes part in DNA synthesis activation loop as a PKC activator [10], catalyzes phosphatidylinositol 4, 5 bisphosphate (PIP2) into diacylglycerol (DAG) and inositol 1, 4, 5-triphosphate (IP3). Since these catalysis products stand for the activation of conventional and novel PKC; atypical PKC might be excluded from these activation loop. Thus, the effect of PKC activators in DNA synthesis might be considered for detection of specific PKC isoforms participated in this process.

Inhibitor of phospholipase C (PLC), that hydrolyses PIP2 is found to significantly reduce PGV (simian virus infected porcine granulosa cell line) cell proliferation. Moreover, inhibitor of phosphatidic acid phosphatase (PAP), that converts phosphatidic acid (PA) to DAG, also significantly reduces PGV cell numbers. PMA restores PGV cell proliferation that is reduced by both PLC and PAP [36]. cAMP independent growth promoting effects of FSH are shown to be activated by calcium ion and MAPK-dependent pathways [37]. These pathways independent of cAMP but dependent of Ca^2+^ and MAPK might include PKC. However, specific PKC isoforms included in these signaling pathways remain unknown.

Research focused on isoform specifity of PKC is very restricted. PKCδ is suggested to be related with anti-apoptotic actions of bFGF in rat granulosa cells [13]. The results of the current study showing that PKCδ is highly expressed in granulosa cells, also support the idea that PKCδ might specifically be critical for inhibition of granulosa cell apoptosis during pre-pubertal, pubertal and adult stages. PKCδ was also found to have specific functions during mouse oocyte maturation [11]. According to our results, PKCδ might also have important roles in granulosa cells. In a recent study, it is suggested that cPKC isoforms PKCα and PKCβI participate in FSH-induced meiotic resumption of mouse follicle enclosed oocytes in follicle culture model [18]. Thus, specific PKC isoforms might be critical for oocyte maturation process. Once, specific PKC isoforms responsible for progression and arrest of oocyte maturation is determined, the mechanisms lying behind oocyte maturation defects can be better understood as well as in vitro maturation of oocytes for treatment and preservation of fertility can be manipulated by the use of specific chemical activators or inhibitors of these isoforms.

PKCε was found to be expressed at a significantly higher level in oocytes of all follicles in pre-pubertal, pubertal and adult ovaries. However, unlike PKCα and PKCδ, PKCε expression was found to be higher in primordial follicles of adult and pre-pubertal ovaries. In PND21 pubertal ovaries, PKCε expression was higher in secondary and pre-antral follicles when compared to primordial and primary follicles. This might be due to differential hormonal levels and related signaling during puberty [28]. PKCε might be critical for primordial follicle survival which is related with balance of oocyte survival and death [38]. The Western blot analysis revealed that PKCε protein expression level was higher in PND60 ovaries when compared to other groups. This might be due to increased number of larger oocytes and elevated expression levels of PKCε in primordial follicles of PND60 ovaries.

During follicular phase, through upstream regulation of FSH and EGF, PKC is found to be included in the activation of DNA synthesis of granulosa cells through induction of MAPK [10]. There is also evidence that the luteolysis process triggered by prostaglandin F_2_α (PGF_2_α) is also under the control of PKC through the activation of Raf/MEK1/ERK1 and ERK2 pathway [22].

PGF_2_α activates PLC through its plasma membrane G-protein-coupled receptor and this activation causes PIP2 hydrolysis and as a result IP3 and DAG accumulation as well as mobilized intracellular Ca^2+^ stimulation [39]. Calcium is required to support progesterone (P4) synthesis in bovine luteal cells and LH increases IP3, and [Ca^2+^]_i_ in bovine luteal cells and in porcine granulosa cells leading to activation of PKC [40]. When PKCε is inhibited with PKCε-specific inhibitors, the PGF_2_α – induced rise in [Ca^2+^]_i_ i is decreased in luteal cells and consequently the ability of PGF_2_α to inhibit LH-stimulated P4 secretion becomes restricted leading to inadequate extension of the luteal life span [41]. On the other hand, inhibition of specific PKC isoform resulting in high P4 level can compensate for luteal phase defects characterized by low P4 levels [42].

Though it is documented that PKCε is related to progesterone synthesis/secretion in luteal cells in earlier studies [23], in the current study PKCα expression was found to be significantly higher in CL compared to the other follicles. These results indicate that PKCα might be important for luteolysis process and luteal functions. PKCδ and PKCε expressions in luteal cells of CL was found to be lower than developing follicles. PKCα must also be studied in terms of its possible functions in luteal cells. Though it is found that PKC family is associated with the MAPK signaling cascade in luteal cells [22], it is still unclear which PKC isoform is specifically related to these signaling pathways in CL.

PKC is found to be participated in the induction process of ovulatory Prkg2 gene through stimulation of ERK, but not JNK [6]. Thus, PKC induces particular genes through interaction with specific proteins. These specific target proteins might be stimulated by specific PKC isoforms. Since, in the current study PKCα and PKCδ expressions were found to be more significant in granulosa cells, signaling pathways related to ovulatory genes might be related to these isoforms.

Though isoform specific PKC antibodies can detect multiple isotypes to some extent, based on our observations in Western blot experiments, we concluded that the antibodies are notably specific for the studied isoforms for Western blot as well as immunohistochemistry.

In addition to our findings through immunohistochemistry and Western blotting, the existence of mRNA of specific PKC isoforms in different cells of the ovary can be revealed by application of q-PCR on isolated cells from follicles at specific developmental stages without any concern about the amount of specimen. Besides, proteomics technology can clearly reveal the structure and function of different PKC isoforms on isolated cells of the ovary.

## Conclusions

We conclude that PKCα, PKCδ and PKCε isoforms show different expression profiles in mouse ovary at pre-pubertal, pubertal and adult stages. Thus, considering the differential expression levels of these isoforms in specific cellular components of the ovary, we suggest that these isoforms have particular roles in ovarian function. The isoform specifity of PKC family must be further studied to reveal undiscovered mechanisms of follicular development, oocyte maturation, ovulation and luteal cell functions. The proof of PKC isoform specifity for particular ovarian functions will allow the use of isoform specific inhibitors or activators to compensate ovarian dysfunction which is one of the major causes of female infertility.
